# Label-Free Extended Gate Field-Effect Transistor for Sensing Microcystin-LR in Freshwater Samples

**DOI:** 10.3390/s25051587

**Published:** 2025-03-05

**Authors:** Sondavid Nandanwar, Songyi Lee, Myeongkee Park, Hak Jun Kim

**Affiliations:** 1 Industry 4.0 Convergence Bionics Engineering, Pukyong National University, Busan 48513, Republic of Korea; david@pknu.ac.kr (S.N.); slee@pknu.ac.kr (S.L.); 2 Department of Chemistry, Pukyong National University, Busan 48513, Republic of Korea; bikeplay@pknu.ac.kr

**Keywords:** EG-FET, DNA aptamer, cyanotoxins, carbon nanotube, microcystin

## Abstract

In this study, we developed a label-free biosensor based on aptamer-modified multi-walled carbon nanotube extended gate field-effect transistor (MWCNT-EG-FET) for easy and selective detection of microcystin-LR (MC-LR), a prominent cyanotoxin associated with liver damage, bleeding, and necrosis. EG-FET had two parts: a MOSFET and an extended-gate Au/SiO_2_ electrode, which serves as the sensitive membrane. A custom-designed DNA oligonucleotide (5-NH_2_-C_6_-AN6) was used as MC-LR-targeting aptamer (MCTA). MWCNTs were functionalized with MCTA and then stably fixed on the sensitive membrane. The detection of MC-LR in freshwater was effectively achieved within 5 min by assessing the variations in electrical resistance that occur due to the selective interactions between MC-LR and MCTA. The detection limit and analytical sensitivity of the biosensor for MC-LR were found to be 0.134 ng/mL and 0.024 ng/mL, respectively. The sensitive membrane could be readily discarded if damaged, eliminating the need to replace the main transducer MOSFET. The developed sensor exhibits features such as straightforward preparation, swift response, potential for miniaturization, and ease of use, making it an attractive candidate for future integrated lab-on-chip systems for MC-LR detection in freshwater environments.

## 1. Introduction

Cyanobacteria, which trace their origins back billions of years, are prevalent in all types of aquatic environments [1]. The eutrophication of lakes and reservoirs is driving the global expansion of harmful cyanobacteria, which increasingly endangers public health globally. Microcystins (MCs) are widely recognized as cyanotoxins associated with algal blooms, and they rank among the most commonly identified toxins in freshwater environments worldwide [2]. Their presence represents a significant risk to both drinking water quality and the health of ecosystems. MCs are structured as cyclic heptapeptides, comprising two variable amino acid positions alongside five constant ones [3]. Owing to its unique circular heptapeptide configuration, this substance exhibits a strong affinity and high specificity for serine/threonine protein phosphatase, thereby serving as a potent inhibitor of the eukaryotic protein phosphatase families PP1 and PP2A, resulting in the induction of cell apoptosis. Moreover, the presence of MCs may instigate oxidative stress in the liver, which could have lethal implications. The variations in toxicity observed among different microcystin strains can be linked to the specific peptide synthase produced by each strain [4,5]. Microcystin-LR stands out as the most toxic form of microcystins, possessing an LD_50_ of 43 µg/kg [6]. The World Health Organization established a guideline value for MC-LR in drinking water in 1998, which was set at 1 µg/L, alongside an acceptable daily intake level of under 0.04 g/kg of body weight [7,8].

Conventional techniques for the detection of MC-LR include high-performance liquid chromatography (HPLC) [9] and enzyme-linked immunosorbent assays (ELISA) [10]. These approaches are esteemed for their outstanding stability and reliability. However, they necessitate controlled laboratory environments, advanced technological tools, expert personnel, and involve sample preparation steps that can be quite complex and time-consuming [11]. These limitations hinder the efficient and swift detection of analytes in actual samples. Thus, it is crucial to devise a method that can swiftly and extensively detect MC-LR in real samples.

Biological sensors are gaining recognition as viable alternatives, marked by their heightened sensitivity, selectivity, user accessibility, and the ability to facilitate continuous monitoring [12]. Recently, there has been a growing emphasis on the advancement of carbon nanotube field-effect transistor (CNT FET) biosensors, attributed to their remarkable sensitivity and rapid response capabilities [13]. The structural characteristics of carbon nanotubes (CNTs), which include an extensive surface area, superior electron mobility, and biocompatibility, position them as excellent substrates for use in biosensor technologies. Field-effect transistors (FETs) effectively convert subtle biological signals into measurable electrical signals, facilitating the detection of analytes that are typically challenging to label without the need for additional markers [14]. Previous research has established the effectiveness of CNT FET biosensors in recognizing various biological entities, including nucleic acids, proteins, and small molecules, without the necessity for labeling, thereby showcasing their significant versatility [15]. The recognition component enables the sensor to accurately identify the target. Aptamers function as biorecognition elements and are composed of single-stranded DNA or RNA oligonucleotides that are selected through in vitro processes [16]. They are recognized for their exceptional specificity, structural stability, ease of synthesis, cost efficiency, and consistent performance across different batches. Consequently, the integration of aptamers into CNT FET biosensors appears to be a promising approach for addressing the ongoing difficulties in detecting cyanotoxins.

Multi-walled carbon nanotubes (MWCNTs) are generally less effective than FET elements, largely due to the fact that the electrical properties of their internal layer tubes do not respond readily to the applied gate field. The issue of intrinsic inefficacy can be addressed through the application of extrinsic functionalization techniques. Our team has developed MWCNTs that have been modified with metallic nanoparticles, such as platinum and gold, to serve as potential active components in FET sensors designed for sugar detection [17]. It is assumed that the electrical conduction mechanism in nanoparticles connected to MWCNTs involves the contact of metallic surfaces, enabling the adsorption of organic chemicals.

In light of this finding, we propose that the electrical conduction pathway of *p*-type conducting MCTA-activated MWCNTs may involve overlapping connections among immobilized MCTAs. Consequently, these bioactivated MWCNTs could serve as highly effective active components in FET devices. In earlier studies, we examined the development of a paper-based immunosensor [18] and top-gated FET [19] that employs biochemically activated MWCNTs for the assessment of microcystin-LR (MC-LR). Both of these sensors showed promising low detection limits for MC-LR.

**Scheme 1:**
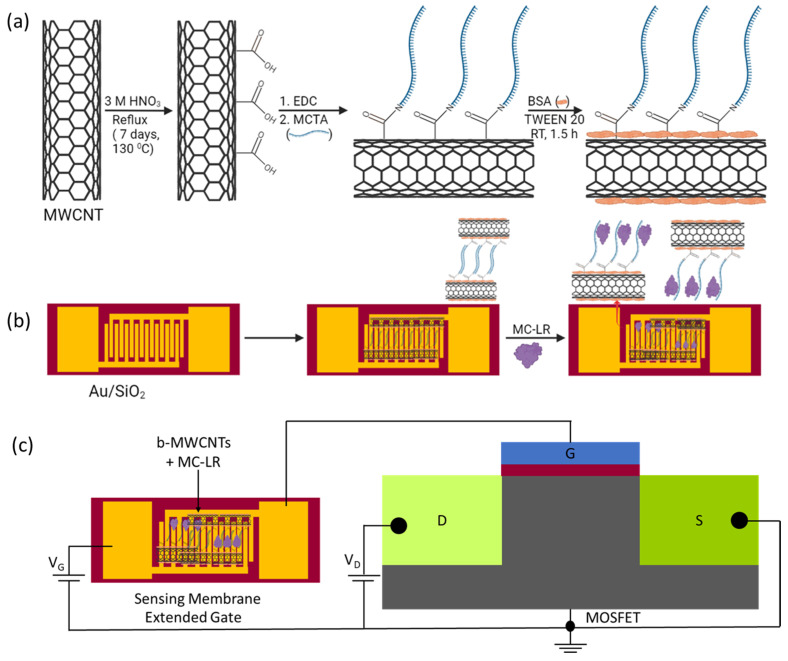
Schematic illustration of extended gate field-effect transistor using functionalized MWCNTs. (**a**) Preparation of b-MWCNTs by linking microcystin-LR targeted aptamers (MCTAs) with carboxylated MWCNTs. (**b**) Deposition of b-MWCNTs on Au/SiO_2_ electrode and interaction with MC-LR. (**c**) Detection of MC-LR using EG-FET biosensor.

This study focused on the development of DNA aptamer-functionalized MWCNT extended gate field-effect transistor (MWCNT EG-FET) (Scheme 1) for the rapid detection of low concentrations of MC-LR in real water samples with enhanced sensitivity and selectivity. The sensor was constructed by fixing MCTA aptamer-modified MWCNTs between two gold-based source and gate electrodes on a silicon dioxide/silicon wafer, forming an ultrathin active channel. The gold electrode was connected to source (S) and gate (G) of MOSFET transducer. The engagement of the aptamers with the target molecule induces electrostatic changes, resulting in a consistent biomolecular response. The MWCNT-EG-FET sensor shows significant potential for ultra-sensitive, label-free, and real-time detection of MC-LR. This advancement paves the way for innovative applications in freshwater quality monitoring, emphasizing its considerable capabilities for on-site testing.

## 2. Materials and Methods

### 2.1. Materials

The multi-wall carbon nanotubes (MWCNTs) were purchased from Carbon Nano-material Tech. Co., Ltd. (Pohang, Republic of Korea). The MWCNTs were produced by chemical vapor deposition (CVD) using iron and molybdenum catalysts. Nitric acid (64.0–65.0%) was obtained from Duksan Science (Ansan, Republic of Korea). 1 M 2-(*N*-Morpholino)ethanesulfonic acid (MES, pH = 5.0), phosphate-buffered saline (PBS, pH 7.4), and 1X PBS buffer (pH 7.4) containing 0.05% Tween 20 and 1% bovine serum albumin (BSA) were purchased from Tech & Innovation (Chuncheon, Republic of Korea). *N*-(3-Dimethylaminopropyl)-*N*′-ethylcarbodiimide hydrochloride (EDC), and gold surface-cleaning solution was purchased from Sigma-Aldrich (St. Louis, MO, USA). MC-LR, MC-LY (L: leucine, Y: tyrosine), and MC-YR (Y: tyrosine, R: arginine) were from Enzo Life Sciences, Inc. (Farmingdale, NY, USA). DNA aptamer (MC-LR targeted aptamer-MCTA: DNA oligonucleotide 5-NH_2_-C_6_-AN6) was synthesized in Macrogen (Seoul, Republic of Korea). Gold microelectrodes with a 10 µm gap, referred to as the source and gate on silicon/silicon dioxide substrate (Au/SiO_2_, 4 mm in width and 6 mm in length), were purchased from DH gate-Hxq315 (Guangzhou, China).

### 2.2. Functionalization of Multi-Walled Carbon Nanotubes

The bioactivation of the MWCNTs was executed based on a method that has been previously described in our earlier research [18]. The MWCNTs underwent carboxylation through treatment with concentrated nitric acid (HNO_3_), as illustrated in Scheme 1. Specifically, 300 mg of MWCNTs were reacted with 150 mL of 3 M HNO_3_ for a duration of seven days at a temperature of 130 °C. Following this reaction, the MWCNTs were thoroughly washed with distilled water through more than ten centrifugation cycles to reach a pH of approximately 7. The carboxylated MWCNTs were bioactivated by forming an amide bond with MCTA. This was achieved by mixing 5.2 mg of carboxylated MWCNTs, 4 µL of EDC, and 2 mL of MCTAs (66 nM) in 16 mL of 0.1 M MES, before allowing the reaction to proceed for 24 h. To avoid unwanted chemical interactions with the surface of MWCNTs, the bioactivated MWCNTs were coated with BSA. This was achieved by treating them with 100 μL of PBS containing 0.05% Tween 20 and 1% BSA. The MWCNTs produced at this stage were referred to as b-MWCNTs, signifying their bioactivated status. Each step of the preparation procedure was followed by an analysis of the modified MWCNTs using Fourier transform infrared spectrometry (FT-IR; JASCO FT/IR-4100, Easton, MD, USA). X-ray photoelectron spectroscopy (XPS; Multilab2000, Thermo Scientific, Waltham, MA, USA) was employed to confirm the chemical environment of the MCTA-attached MWCNTs. The XPS profiles of the samples were analyzed through deconvolution to verify bioactivation, with a primary focus on the FT-IR spectra discussed in our earlier research [18]. The stock solution of b-MWCNTs was prepared in distilled water at a concentration of 1 mg/mL and kept in refrigerator for further use.

### 2.3. Fabrication of MWCNT-Based EG-FET Device

**Figure 1:**
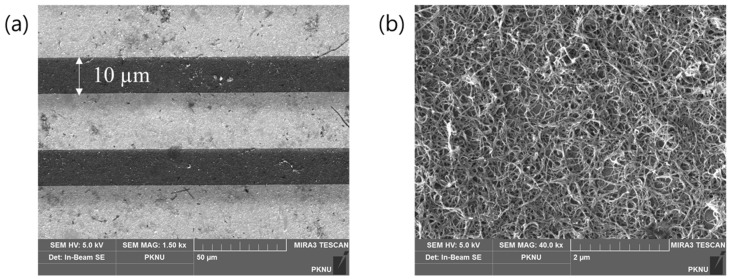
SEM images of the (**a**) b-MWCNTs immobilized between the source and gate electrodes by solution dropping and immobilized b-MWCNTs after washing with PBS (scale bars: 50 µm), and (**b**) b-MWCNTs deposited on the surface of the electrode.

The proposed EG-FET sensor consists of two parts: the MOSFET transducer and a sensing extended gate electrode. The Au/SiO_2_ microelectrode with a 10 µm wide gap were used as an extended gate electrode. The sensing extended gate electrode was prepared as follows: The Au/SiO_2_ microelectrode was cleaned by dipping it in a gold cleaning solution for 5 s, followed by washing with distilled water, and drying under inert condition (99.99% N_2_, Hanagas, Gimhae, Republic of Korea). The stock solution of b-MWCNTs was diluted to a concentration of 0.1 mg/mL and sonicated for 30 min. One microliter of the b-MWCNT suspension was dropped on the cleaned Au/SiO_2_ microelectrodes and subsequently dried in a convection oven at 50 °C for 5 min to fix the b-MWCNTs between the source (S) and gate (G) electrode fingers (Scheme 1b). The b-MWCNTs-Au/SiO_2_ electrode was analyzed using scanning electron microscopy (SEM, Hitachi S2400, Tokyo, Japan, Figure 1). The b-MWCNTs-Au/SiO_2_ was fixed on the adaptor plate. Next, the microelectrode was connected to S of MOSFET transducer using copper wire and connected to the gate (G) using silver wire and silver paste. These EG-FETs offer notable advantages due to their requirement for a considerably smaller gate voltage range (*V*_G_~±1 V), especially in comparison to the ±20 V necessary for back-gating operations [12]. Additionally, EG-FET sensors typically demonstrate a sensitivity that is around ten times higher than that of back-gated FET sensors. The developed sensor was reused for the detection of MC-LR. The fixed b-MWCNTs-Au/SiO_2_ electrode was removed from the adapter and carefully cleaned used Au cleansing solution and thoroughly washed with distilled water before the sensor was refabricated for reuse.

### 2.4. Electrical Measurement

The varying concentrations (0–2.5 ng/mL) of MC-LR were prepared in the 1 mM PBS using the MC-LR stock solution, with 1 µL of MC-LR stock solution dropped on the b-MWCNTs-Au/SiO_2_ microelectrode. Subsequently, the *I*_ds_-*V*_ds_ characteristics (with a constant *V*_gs_ of 1 V) and *I*_ds_-*V*_gs_ characteristics (with a constant *V*_ds_ of 1 V) of the biosensor were instantaneously measured using a computer interface connected to a constant current source (digital multimeter; Keithley 196) and a DC power source (T7 with LJTick-DAC, Labjack, Lakewood, CO, USA). Unless otherwise specified, all measurements were performed at room temperature.

The *I*_ds_ versus *V*_gs_ plots were utilized to compute the relative transconductance (*g*_m_/*g*_mo_), sensitivity (*R*/*R*_sat_), and limit of detection (LOD). The equation *g*_m_ = μ*_h_*(*C*/*L*^2^)*V*_ds_ (μ*_h_* represents mobility, *C* denotes nanotube capacitance, *L* signifies nanotube length, and *V*_ds_ corresponds to the voltage applied between the source and drain electrodes) was used to calculate the change in *I*_ds_ for a given change in *V*_gs_ (*g*_m_ = *I*_ds_/*V*_gs_) above the threshold voltage (*V*_TH_) at a constant *V*_ds_ [20]. The LOD was determined as follows: LOD = (y − b)/a = (y0 + 2SD − b)/a, where a is the slope of the linear fitting line, b is the intercept, and SD is the standard deviation of the signal at 0.0 ng/mL of MC-LR.

Additionally, the *I*_ds_ values were recorded over time at different concentrations of MC-LR, and the change in *I*_ds_ (Δ*I*_ds_) was noted. Commercially available mineral water (FW), laboratory tap water (TW), filtered tap water from a drinking fountain (FTW, filtered using a sub-micropore filter with a pore size of 0.04 μm), and filtered Yeongju dam water (FYW, filtered with a pore size of 0.45 μm) [18] were employed as real water samples for the experiment. The *I*_ds_-*V*_gs_ characteristics were measured for MC congeners (MC-YR and MC-LY; 0–2.5 ng/mL) alone, and MC-LR (0–2.5 ng/mL) in the presence of MC congeners (1.5 ng/mL). The *I*_ds_ versus *V*_gs_ plots for MC congeners alone and MC-LR in the presence of additional MC congeners were used to calculate relative transconductance (*g*_m_/*g*_mo_).

## 3. Results

### 3.1. Characteristics

**Figure 2:**
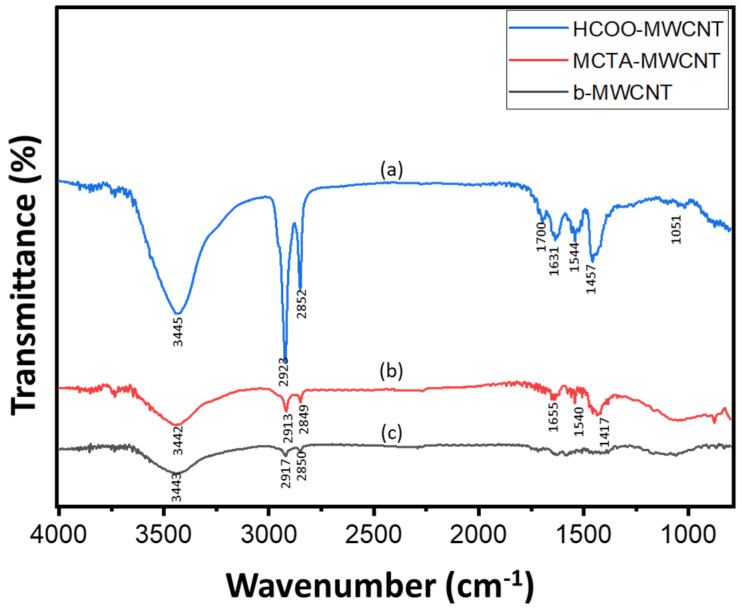
FT−IR spectra of (**a**) carboxylated MWCNTs, (**b**) MCTA attached MWCNTs, and (**c**) BSA−coated MCTA-MWCNTs (b-MWCNTs).

Figure 2 presents the FT-IR spectrum of modified MWCNTs. In spectrum (a), the peak at 1530–1560 cm^−1^ is assigned to stretching mode of the C=C double bond that forms the framework of the carbon nanotube sidewall [21]. The appearance of the absorption peaks at 1700 and 1051 cm^−1^ in the IR spectra of MWCNT-COOH clearly indicates carboxylic groups on the MWCNTs [22]. The two bands at 2800–3000 cm^−1^ which are seen in all spectra are assigned to CH stretching of MWCNT-COOH defects [18]. In the spectrum (b), the appearance of the peak at 1540 cm^−1^ (NH stretching modes) and 1655 cm^−1^ (C(=O)NH linkage) indicates that MCTA has been successfully attached onto the external surface of MWCNTs. From these results, it can be deduced that the formation of amide bonds can lead to the strong linkage of MCTA with carboxylic groups of MWCNT-COOH. The IR spectrum of b−MWCNTs (Figure 2c) shows that the intensity of the characteristic bands reduced by the BSA coating [18].

**Figure 3:**
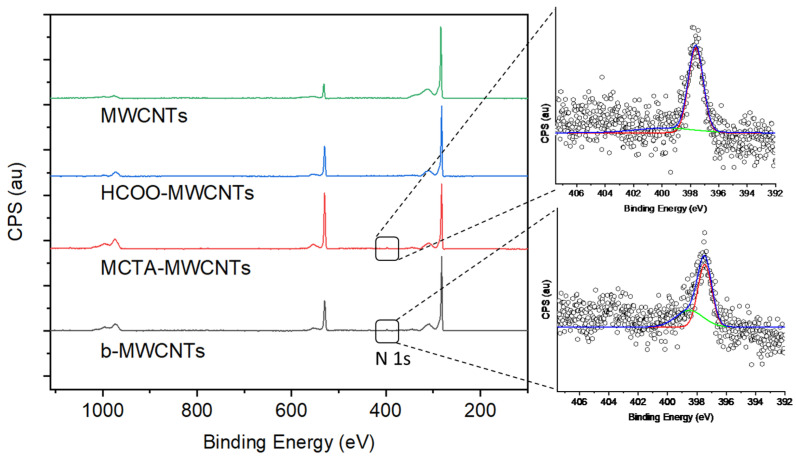
XPS spectrum of MWCNTs, HOOC-MWCNTs, MCTA-MWCNTs, and b-MWCNTs. The right-side plot is the N 1s band.

**Figure 4:**
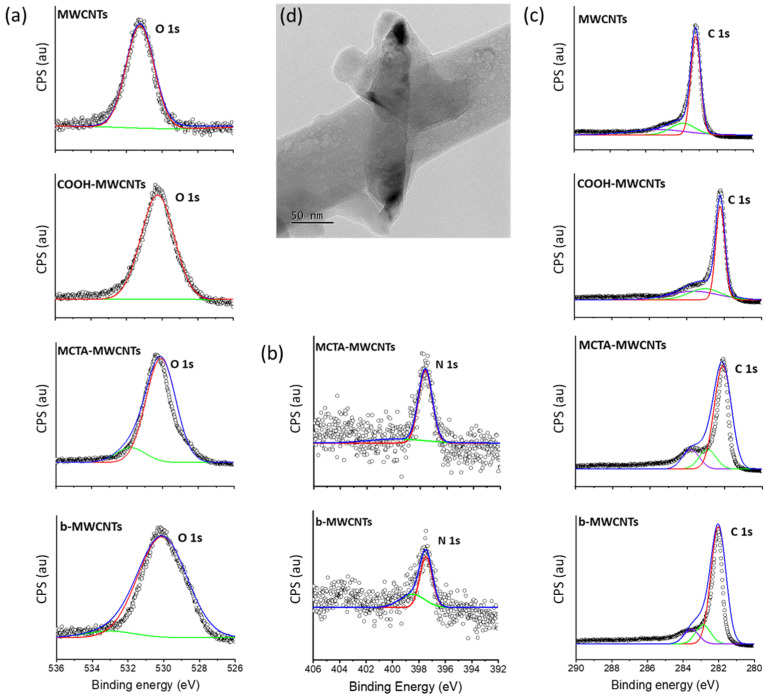
X-ray photoelectron spectroscopy (XPS) profiles near binding energy of (**a**) oxygen, (**b**) nitrogen, and (**c**) carbon for the functionalized and pristine MWCNTs. The acquisition of high-resolution N 1s spectra for MWCNTs and COOH-MWCNTs was not possible because the survey spectrum did not reveal any detectable signals. (**d**) Transmission electron microscopy (TEM) image of the MCTA-MWCNT filaments with a width of ~100 nm.

The bioactivation of the MWCNTs was confirmed by XPS analysis, as shown in Figure 3 and Figure 4. The XPS data demonstrated the variations in the chemical properties of the CNT surface observed before and after the functionalization. The O 1s band of the MWCNTs exhibits a notably weak intensity, as illustrated in Figure 4a, primarily due to the presence of adventitious carbon contamination. Upon the carboxylation of MWCNT (COOH-MWCNTs), the O 1s band exhibits a slight increase in binding energy. A notable increase in the intensity of the O 1s band was observed in the MCTA-MWCNTs and b-MWCNTs, which can be ascribed to the oxygen atoms formed in the MCTAs and BSAs. The N 1s band observed in these samples, as indicated in Figure 4b, originates from MCTAs and BSA. The N 1s band at 400 eV is associated with nitrogen in the amide (O=C–N–) group, while the shoulder peak observed around 402 eV is indicative of protonated nitrogen within the same amide group [23]. In Figure 4c, the shoulder peak of the C 1s band, associated with oxygenated carbon species, exhibited an enhancement and a shift to a higher energy level due to bioactivation. The findings validate the process of bioactivation and are consistent with the previously described FT-IR analysis [18]. The TEM image of the bioactivated MWCNTs is presented in Figure 4d. The diameter of the MCTA-MWCNTs closely resembles that of the pristine MWCNTs.

### 3.2. Sensor Sensitivity

**Figure 5:**
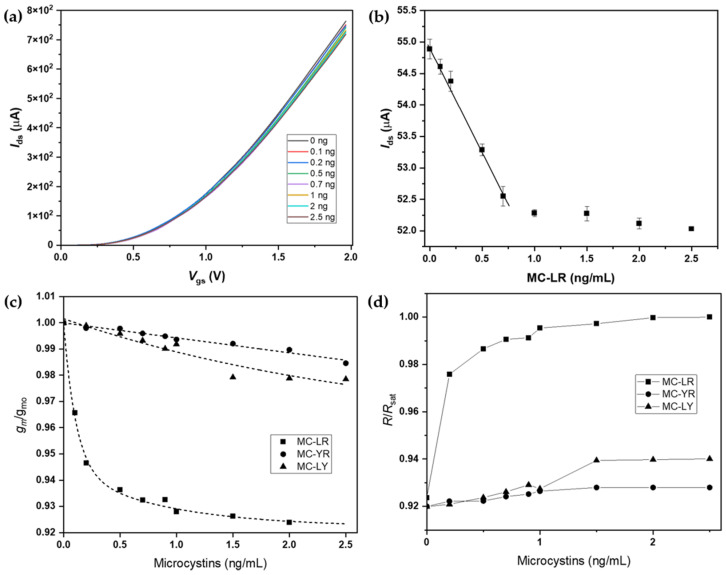
(**a**) *I*_ds_ vs. *V*_gs_ plot at constant *V*_ds_ = 1, (**b**) change in *I*_ds_ with the increase in the concentration of MC-LR (0 and 2.5 ng/mL) in PBS, (**c**) relative transconductance of the biosensor for MC-LR, MC-YR, and MC-LY, and (**d**) sensitivity (*R*/*R*_sat_) of the biosensor for the MC-LR, MC-YR, and MC-LY in PBS.

The developed EG-FET biosensor was used for the detection of MC-LR under different conditions. The *I*_ds_ vs. *V*_gs_ plot for the increasing concentration of MC-LR (0–2.5 ng/mL) is shown in Figure 5a. The *I*_ds_ values dropped when the MC-LR concentrations increase (Figure 5b), revealing that *I*_ds_ and MC-LR have an inverse relationship. The *I*_ds_ values drop linearly until the concentration of MC-LR reaches up to 1 ng/mL without moving *V*_TH_ = 0.1 V, which begins to saturate as the concentration of MC-LR increases. These results show that the developed biosensor could detect up to 1 ng/mL of MC-LR at maximum concentration.

The *I*_ds_ vs. *V*_gs_ plot at constant *V*_ds_ = 1 (Figure 5a) is used to calculate the relative transconductance (*g*_m_/*g*_mo_) of the EG-FET biosensor against increasing concentrations of MC-LR (0–2.5 ng/mL) (Figure 5b,c). An increase in MC-LR concentrations leads to a decline in the *g*_m_/*g*_mo_ values. When MC-LR and MCTA interact, the conduction channel produced by MCTA might be disrupted, resulting in a decrease in conductance or an increase in resistance of the biosensor. The sensor shows a linear increase in sensitivity at a low concentration (0.2–1 ng/mL) of MC-LR in 1 mM PBS. The analytical sensitivity of the biosensor, which is estimated from the slope of the linear range, is 0.024 ng/mL for MC-LR. The LOD of the biosensor is 0.134 ng/mL. The analytical sensitivity of the developed biosensor is 7.5 times higher, whereas the LOD is slightly improved compared to our previous paper-based biosensor [18]. The efficacy of the EG-FET sensor developed in this research was evaluated against other electrochemical sensors known for their high sensitivity, as presented in Table 1. The developed EG-FET sensor showed higher sensitivity for MC-LR compared to the SPCE/CS sensor [24], whereas lower sensitivity compared to sensors such as AgI–TiO_2_/DNA [25], AuNPs/Apt/NaCl [26], GQDs/SiNWs [27], Au/MB/Apt [28], MXene/cDNA-MB [29], and LIG/MB/Apt [30]. Nevertheless, the detection limit of the proposed EG-FET sensor was sufficiently lower for practical applications.

The sensitivity (*R*/*R*_sat_) of the biosensor (Figure 5d) is determined from the data obtained from the *I*_ds_ vs. *V*_gs_ plot at constant *V*_ds_ = 1 (Figure 5a). The resistance is calculated using Ohm’s law equation, using the values of *I*_ds_ and *V*_gs_. Figure 5d outlines the relative transconductance values of the EG-FET as a function of MC congeners (MC-LR, MC-YR, and MC-LY) in PBS solution at fixed *V*_ds_ = 1 V and *V*_gs_ = 1 V (Figures S1 and S2). The microcystin congeners MC-LR, MC-YR, and MC-LY are typically present in freshwater ecosystems [31]. An increase in concentrations of MC congeners leads to a decline in the *g*_m_/*g*_mo_ values. Although MC-LR, MC-YR, and MC-LY share significant structural similarities, the EG-FET sensor exhibited a marked sensitivity for MC-LR within the tested concentration range. Both MC-YR and MC-LY displayed relatively smaller change in *g*_m_/*g*_mo_, while *g*_m_/*g*_mo_ showed a consistent decline as the concentration of MC-LR increased. This finding demonstrates the targeted response of the constructed EG-FET sensor towards MC-LR when compared to its congeners.

**Figure 6:**
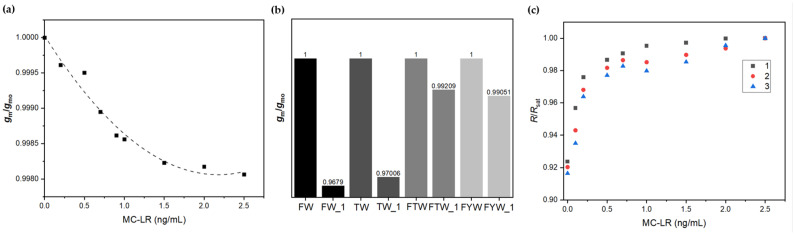
(**a**) Relative transconductance of the biosensor for MC-LR in the presence of other MC congeners (1.5 ng/mL). (**b**) Relative transconductance of the biosensor for MC-LR in MW, TW, FTW, and FYW. The MW, TW, FTW, and FYW contain 0 ng/mL MC-LR, while MW_1, TW_1, FTW_1, and FYW_1 contain 0.75 ng/mL MC-LR. (**c**) Reusability of the sensor.

Figure 6a outlines the relative transconductance values of the EG-FET as a function of MC-LR in PBS solution containing 1.5 ng/mL of MC-YR and MC-LY at fixed *V*_ds_ = 1 V and *V*_gs_ = 1 V (Figure S3). The EG-FET sensor shows the selective detection capability for various MC-LR concentrations in the presence of their congeners, indicating it could selectively detect the MC-LR in real water samples. Further, we checked the MC-LR sensing ability of the developed EG-FET sensor in real water samples such as commercially available mineral water (MW), laboratory tap water (TW), filtered tap water from a drinking fountain (FTW, filtered using a sub-micropore filter with a pore size of 0.45 μm), and filtered Yeongju dam water (FYW, filtered with a pore size of 0.45 μm) (Figure 6b). The solutions of MC-LR (0.75 ng/mL) were spiked in real water (MW, TW, FTW, and FYW) and their *g*_m_/*g*_mo_ was measured at fixed *V*_ds_ = 1 V and *V*_gs_ = 1 V (Figure S4). The EG-FET demonstrated greater sensitivity to MC-LR in both FW and TW, while exhibiting reduced sensitivity in FTW and FYW. Despite the reduced sensitivity for MC-LR observed in FTW and FYW, the data suggest that the developed EG-FET sensor is capable of selectively detecting MC-LR in actual water samples. Consequently, the established bioactivated MWCNT-EG-FET sensor is an efficient, user-friendly, cost-effective, and reliable environmental sensor for the detection of MC-LR in freshwater systems. The sensor’s capability for reusability was validated by the refabrication process that incorporated the reuse of the Au-SiO_2_ electrode. The *I*_ds_ vs. *V*_gs_ plot at constant *V*_ds_ = 1 for second and third repetition is shown in Figures S5 and S6. The EG-FET sensor demonstrated limit of detection (LOD) values for MC-LR of 0.166 ng/mL in the second repetition and 0.175 ng/mL in the third repetition (Figure 6c). The EG-FET sensor exhibited a minor reduction in the limit of detection (LOD) upon reuse; however, the values remain satisfactory for the identification of MC-LR.

## 4. Conclusions

A bioactivated MWCNT-based label-free extended gate field-effect transistor (EG-FET) sensor was developed to detect microcystin-LR (MC-LR). The utilization of the aptamer as the recognition element made our sensor highly sensitive, selective, and stable. Our EG-FET sensor showed a unique sub-nanogram detection limit and high selectivity over a wide range of MC-LR concentrations. The biosensor could easily detect MC-LR from 0 to 1.0 ng/mL in PBS buffer within 5 min without the need for expensive and sophisticated instrumentation. Its higher analytical sensitivity and low detection limit (0.024 ng/mL and 0.134 ng/mL) make it a promising candidate for the detection of MC-LR in real water samples. This biosensor worked faster and showed higher sensitivity towards MC-LR than our previously developed biosensor (MCTA-modified MWCNTs on filter paper). In addition, the sensor showed good selectivity for MC-LR in the presence of structurally similar MC congeners and high sensitivity, enabling the detection of MC-LR within the WHO guidelines for drinking water (≤1.0 ng/mL). Even though the detection performance of this simple and inexpensive biosensor is below that of LC/MS/MS, it shows potential for monitoring MC-LR in real water samples at any remote sites without the need for qualified personnel.

## Figures and Tables

**Table 1:** Comparison of analytical performance of the MCTA-MWCNT with other sensors for detection of microcystin-LR ^1^.

Sensors	Detection Techniques	Linear Ranges	LOD	References
AgI–TiO_2_/DNA	OPECT	0.1 fg/mL–10 pg/mL	0.079 fg/mL	[25]
AuNPs/Apt/NaCl	Colorimetry	0.5 ng/L–7.5 μg/L	0.37 ng/L	[26]
GQDs/SiNWs	PEI	0.1–10 μg/L	0.055 μg/L	[27]
Au/MB/Apt	E-AB	1–750 ng/L	0.53 ng/L	[28]
SPCE/CS	EIS	0.1–100 μg/L	0.69 μg/L	[24]
MXene/cDNA-MB	E-AB	0.0001–5 ng/L	4 × 10^−5^ ng/L	[29]
LIG/MB/Apt	E-AB	10^−2^–10^5^ pg/L	3 × 10^−3^ pg/L	[30]
MWCNTs/Apt	EG-FET	0.1–1 ng/L	0.139 µg/L	In this study

^1^ Abbreviations: Organic photoelectrochemical transistor, OPECT; gold nanoparticles, AuNPs; aptamer, Apt; graphene quantum dots, GQDs; silicon nanowires, SiNWs; electrochemical aptamer-based, E-AB; methylene blue, MB; screen-printed carbon electrode, SPCE; cysteamine, CS; electrochemical impedance spectroscopy, EIS; laser-induced graphene, LIG; complementary DNA, cDNA).

## Data Availability

Data are contained within the article.

## Supplementary Materials

The following supporting information can be downloaded at: https://www.mdpi.com/article/10.3390/s25051587/s1, Figure S1. The current characteristic curves in the presence of varying concentrations of MC-LY solution (0–2.5 ng/mL) at constant *V*_ds_ = 1. Figure S2. The current characteristic curves in the presence of varying concentrations of MC-YR solution (0–2.5 ng/mL) at constant *V*_ds_ = 1. Figure S3. The current characteristic curves in the presence of varying concentrations of MC-LR (0–2.5 ng/mL) in 1.5 ng/mL MC-LY + 1.5 ng/mL MC-YR solution mixture at constant *V*_ds_ = 1. Figure S4. The current characteristic curves in the presence of varying concentrations of MC-LR (0–2.5 ng/mL) in 1.5 ng/mL MC-LY + 1.5 ng/mL MC-YR solution mixture at constant *V*_ds_ = 1. Figure S5. The current characteristic curves (2nd repetition) in the presence of varying concentrations of MC-LR (0–2.5 ng/mL) at constant *V*_ds_ = 1. Figure S6. The current characteristic curves (3rd repetition) in the presence of varying concentrations of MC-LR (0–2.5 ng/mL) at constant *V*_ds_ = 1.
