# Supplementary material for: Label-Free Extended Gate Field-Effect Transistor for Sensing Microcystin-LR in Freshwater Samples
Source: Sensors (Basel). 2025 Mar 5;25(5):1587. doi: 10.3390/s25051587 (PMC11902771; doi:10.3390/s25051587)
Supplement: Supplementary file 1 [file sensors-25-01587-s001.zip › sensors-3494547-supplementary.pdf]

Supplementary Materials

Label-free Extended Gate Field-Effect Transistor for Sensing Microcystin-LR in Freshwater Samples

Sondavid Nandanwar [1], Songyi Lee[1,2], Myeongkee Park[2] and Hak Jun Kim [2,*]

[1] Industry 4.0 Convergence Bionics Engineering, Pukyong National University, Busan 48513, Korea; sondavidknandanwar@gmail.com

[2] Department of Chemistry, Pukyong National University, Busan 48513, Korea.

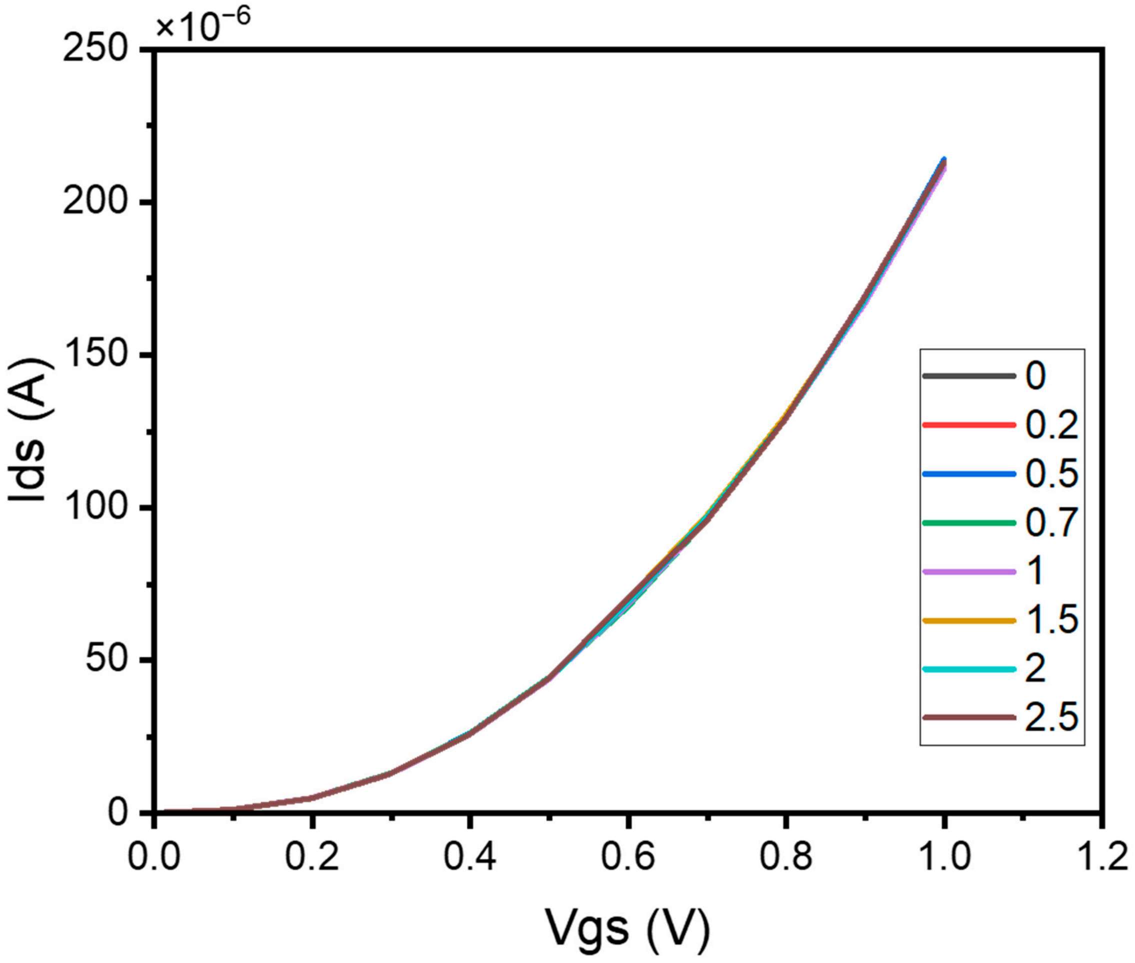

Figure S1. The current characteristic curves in the presence of varying concentrations of MC-LY solution (from 0 – 2.5 ng/mL) at constant V_{ds} = 1.

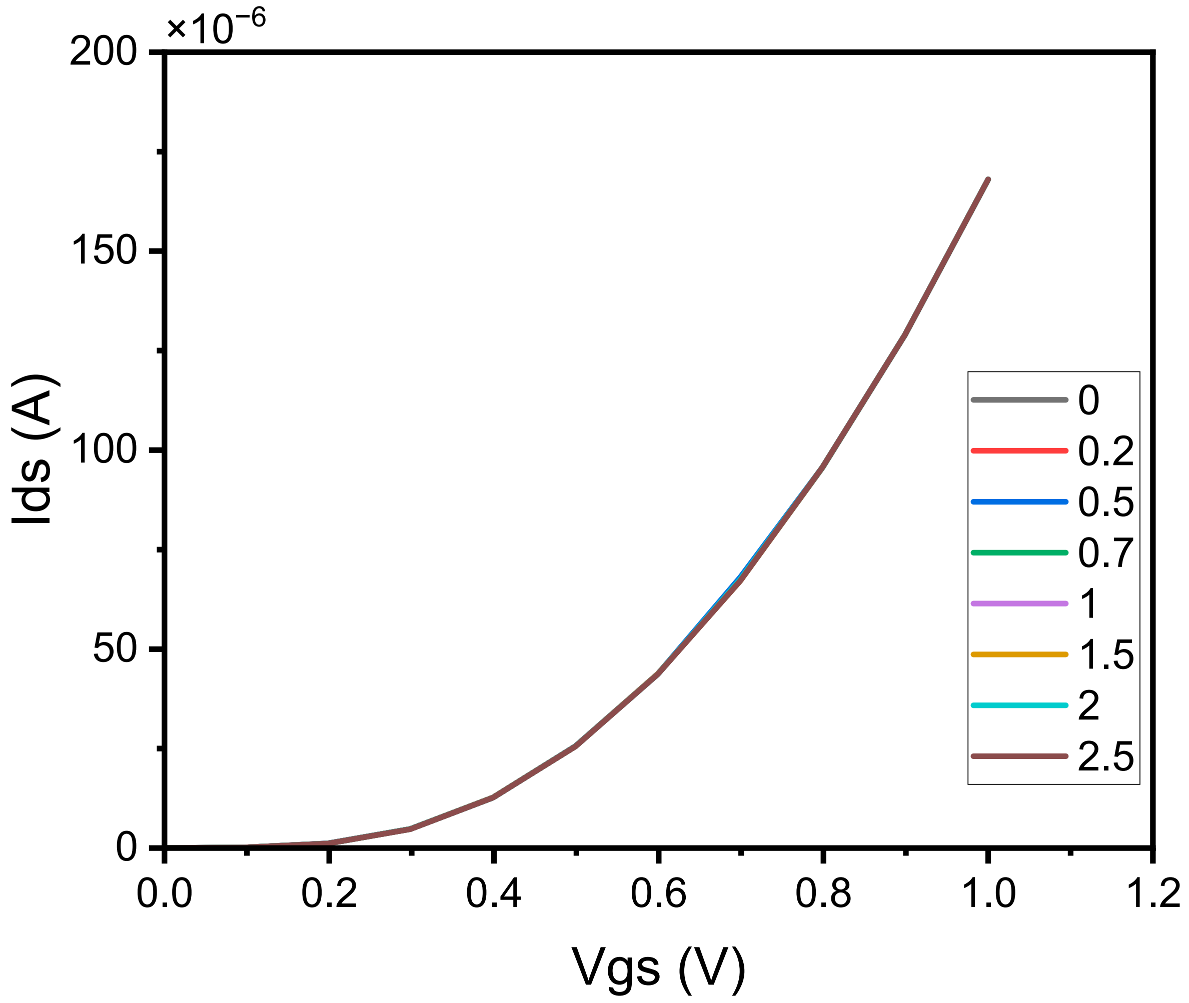

Figure S2. The current characteristic curves in the presence of varying concentrations of MC-YR solution (from 0 – 2.5 ng/mL) at constant V_{ds} = 1.

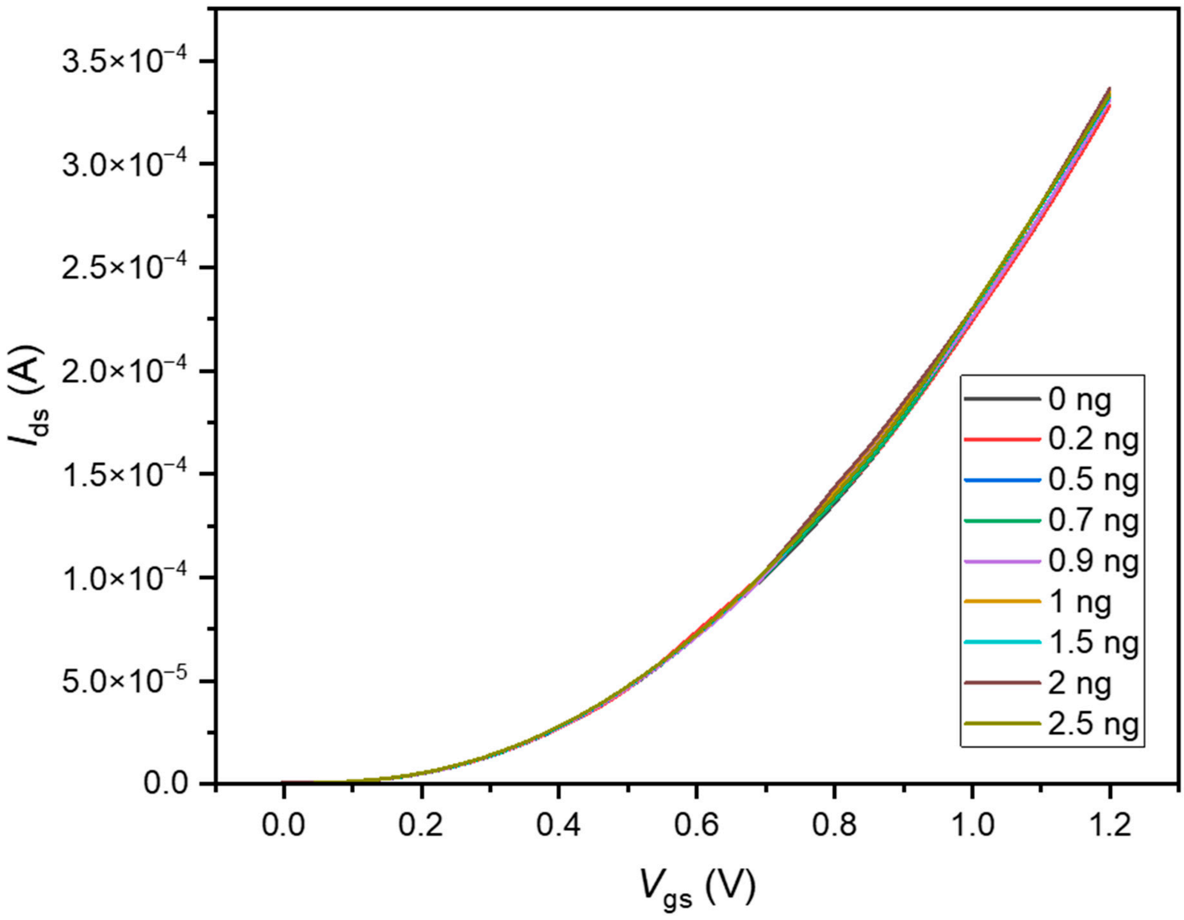

Figure S3. The current characteristic curves in the presence of varying concentrations of MC-LR (from 0 – 2.5 ng/mL) in 1.5 ng/mL MC-LY + 1.5 ng/mL MC-YR solution mixture at constant V_{ds} = 1.

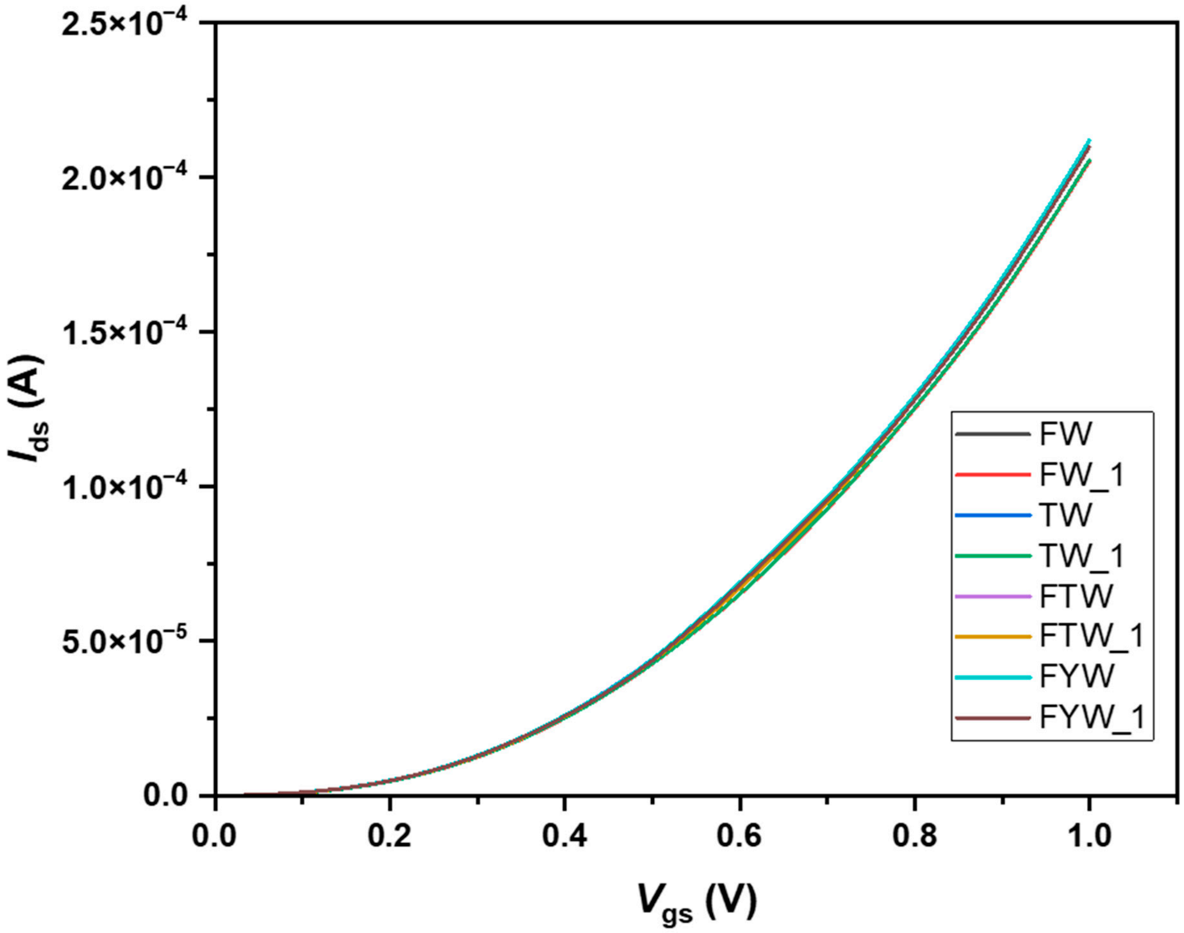

Figure S4. The current characteristic curves in the presence of varying concentrations of MC-LR (from 0 – 2.5 ng/mL) LR in MW, TW, FTW, and FYW. The MW, TW, FTW, and FYW contain 0 ng/mL MC-LR, while MW_1, TW_1, FTW_1, and FYW_1 contain 0.75 ng/mL MC-LR at constant V_{ds} = 1 and V_{gs} = 1V.

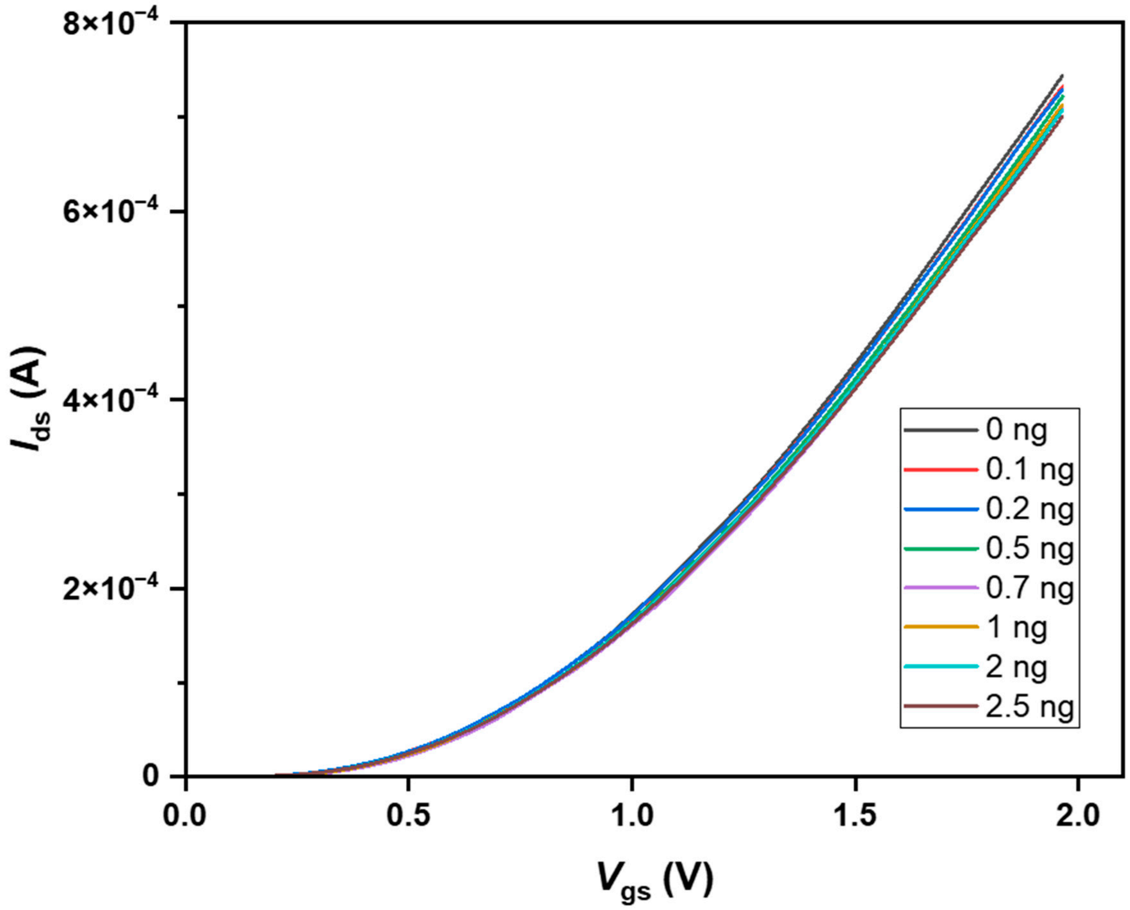

Figure S5. The current characteristic curves (2 nd repetition) in the presence of varying concentrations of MC-LR (from 0 – 2.5 ng/mL) at constant V_{ds} = 1.

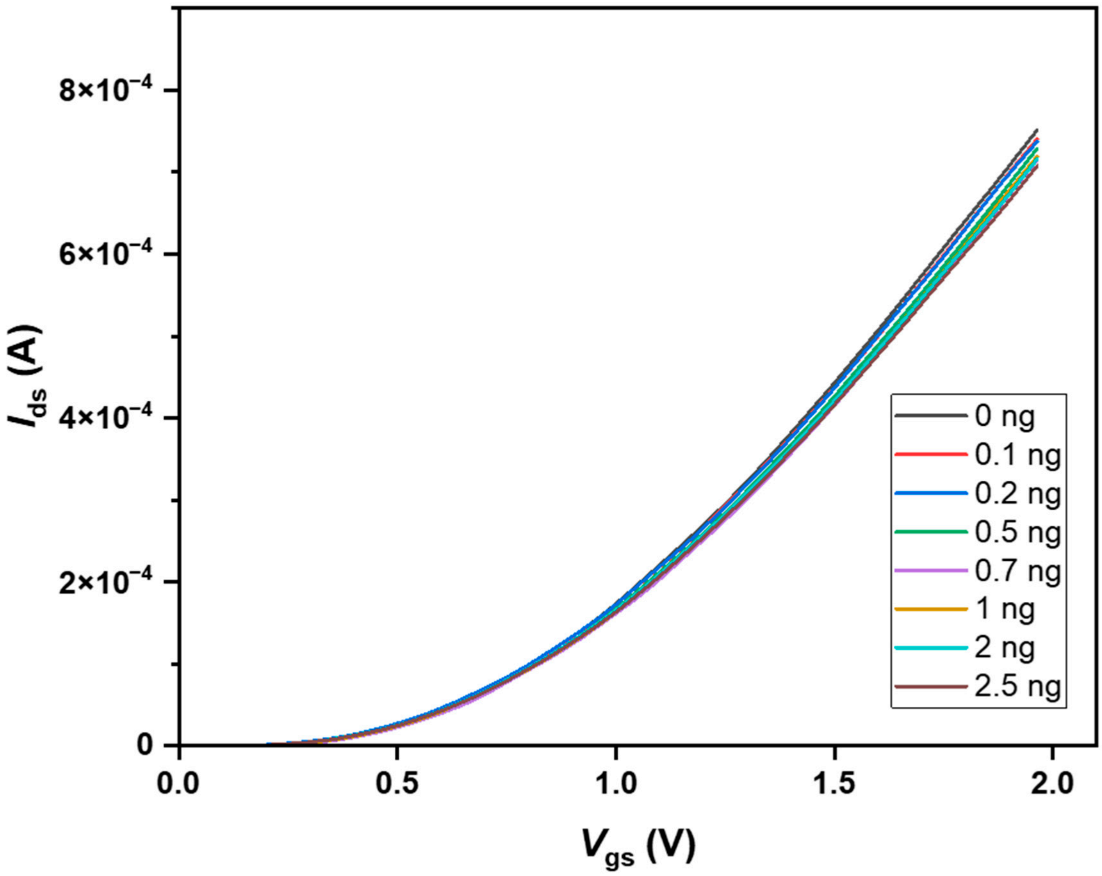

Figure S6. The current characteristic curves (3 rd repetition) in the presence of varying concentrations of MC-LR (from 0 – 2.5 ng/mL) at constant V_{ds} = 1.
